# Monthly and Diel Acoustic Occurrence of Four Baleen Whale Species in South African Waters

**DOI:** 10.1002/ece3.72004

**Published:** 2025-08-18

**Authors:** Fannie W. Shabangu, Kuhle Hlati, Marcel A. van den Berg, Tarron Lamont, Stephen P. Kirkman

**Affiliations:** ^1^ Fisheries Management Branch Department of Forestry, Fisheries and the Environment Foreshore Cape Town South Africa; ^2^ Mammal Research Institute Whale Unit, Department of Zoology and Entomology University of Pretoria Hatfield Pretoria South Africa; ^3^ Oceans and Coasts Branch Department of Forestry, Fisheries and the Environment Foreshore Cape Town South Africa; ^4^ Department of Botany and Zoology Stellenbosch University Stellenbosch Cape Town South Africa; ^5^ Department of Oceanography and Nansen–Tutu Centre for Marine Environmental Research University of Cape Town Rondebosch Cape Town South Africa; ^6^ Bayworld Centre for Research and Education Constantia Cape Town South Africa; ^7^ Institute for Coastal and Marine Research Nelson Mandela University Gqeberha South Africa

**Keywords:** acoustic ecology, acoustic repertoire, ecosystem modelling, environmental variables, low latitude, marine mammals, movement

## Abstract

Understanding of the spatio‐temporal occurrence of cetaceans post the whaling era is essential for protecting and improving management strategies of these marine mammals. To determine the monthly and diel acoustic occurrence of four baleen whale species relative to environmental conditions off the west coast of South Africa, we collected passive acoustic monitoring data within Child's Bank marine protected area in January and May through October 2024 at various water depths. Burst tonal calls of the southern African Bryde's whale offshore population were detected in January and May through July with the highest occurrence in January. Humpback whale songs and southern right whale gunshot sounds were detected from May through October with high occurrence in September and with smaller modes in other months. Antarctic minke whale bioduck calls were also found in June through October, showing high occurrence in August through October. Calls from an unknown source with similar characteristics to Antarctic minke whale bioduck calls were present in May, July, and August with the highest occurrence in August. Diel acoustic occurrence of Bryde's, southern right, Antarctic minke, and minke‐like whale calls indicated that these animals vocalised more during the day while humpback whales were more vocally active at night. Sea surface height and sea surface temperature, either separately or in combination, were the most important predictors of whale acoustic occurrence, highlighting the influence of environmental conditions on the distribution, habitat selection, and ecology of these whales. Overall, this study advances our understanding of the movement, occurrence, and behavioural patterns of several baleen whales relative to environmental conditions. It also provides the first description of the southern African Bryde's whale offshore population's call characteristics, which will be useful at guiding future studies to acoustically differentiate between it and the inshore population.

## Graphical abstract

This study shows that Bryde's, humpback, Antarctic minke and southern right whales occur monthly in South African waters; their occurrence coincides with changes in environmental conditions. We highlight the west coast of South Africa as an important habitat of these whales.

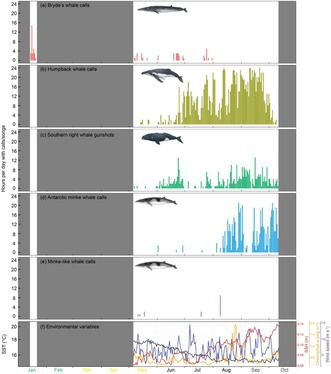

## 1 Introduction

Up‐to‐date information on seasonal occurrence and distribution of marine species such as whales is important and necessary to properly protect and conserve these animals from anthropogenic activities that are on the increase in the ocean. However, vital life history details on seasonal occurrence and distribution of whale species are limited in many regions of the world. For example, in the southern African subregion including South Africa, life history knowledge on whales has mainly been based upon limited stranding and sighting data and historical whaling records (e.g., Best 2007; Elwen et al. 2011). Such data contributed to the understanding of migratory habits of baleen whales that frequent the coastal and offshore waters of South Africa, such as southern right whales ( *Eubalaena australis* ), humpback whales ( *Megaptera novaeangliae* ) and Antarctic minke whales ( *Balaenoptera bonaerensis* ), which typically perform long migrations between low latitude breeding and overwintering grounds and summer foraging grounds in the Antarctic and sub‐Antarctic regions (e.g., Best 2007; Mate et al. 2011; Vermeulen et al. 2024). Even so, it has become known that groups or individuals of these species may forego the long migrations to the Southern Ocean, remaining year‐round in lower latitude areas—this may be due to the suitability of habitats and availability of prey in lower latitude areas allowing for overall energy conservation by remaining there (Best et al. 1995; Barendse and Best 2014; Barendse et al. 2011, 2013; Shabangu, Findlay, and Stafford 2020). Another species found off South Africa's coast, Bryde's whales ( *B. edeni* ), has a tropical and subtropical distribution and does not perform long migrations to Antarctica; instead, they seasonally conduct regional migrations in the waters of the southern African subregion (Best 1996, 2001).

**FIGURE 1:**
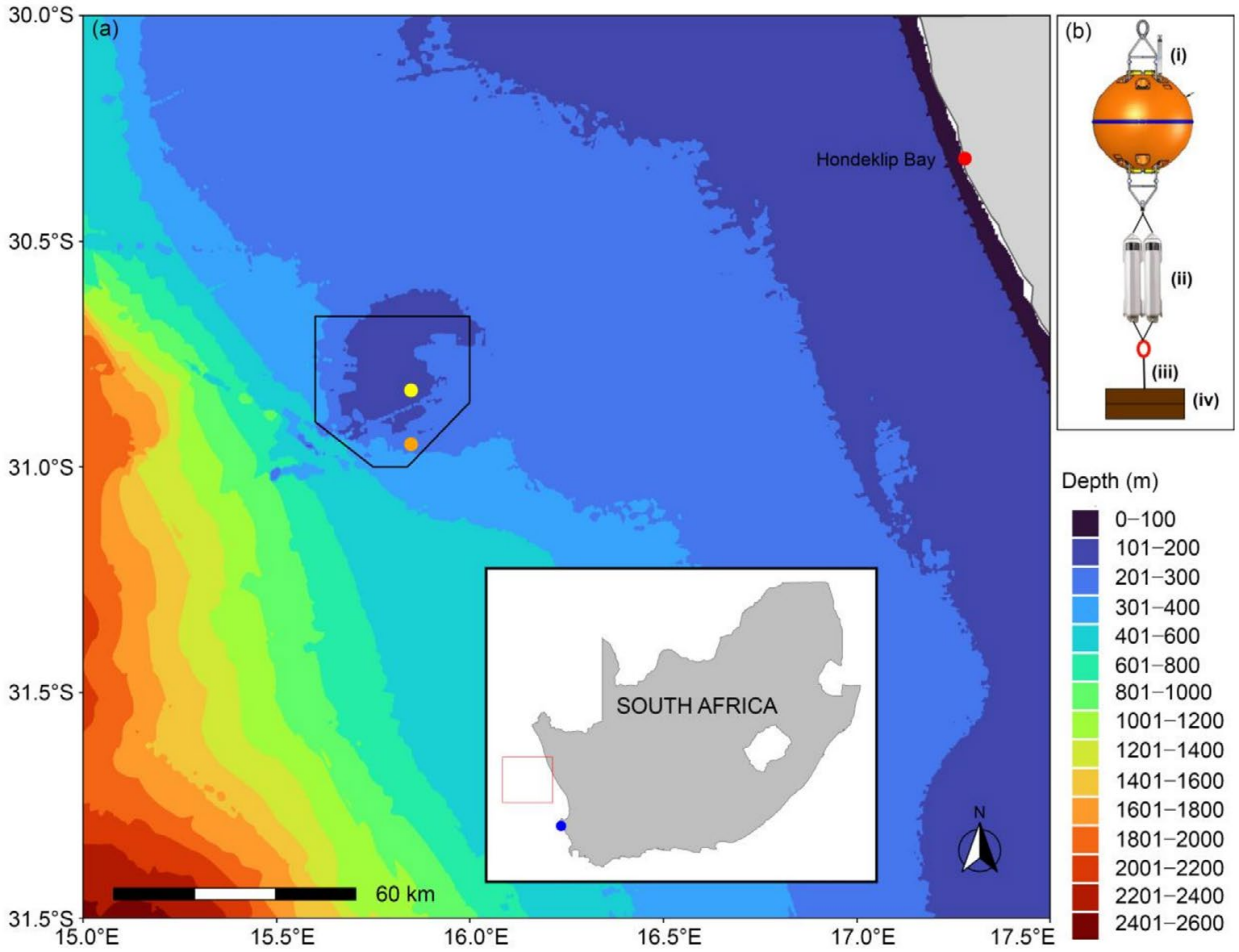
(a) Location of the January (orange circle) and May–October (yellow circle) acoustic mooring within Child's Bank MPA (black hexagon) on the west coast of South Africa (inset map). (b) Schematic of the 5 m tall acoustic mooring consisting of (i) SoundTrap acoustic recorder deployed on a 36″ float, (ii) two Teledyne Benthos R2K acoustic releases, (iii) chain weaved with a rope linked to the oblong (open red circle) and (iv) mooring anchor made of railway tracks weighing around 430 kg. The location of Donkergat whaling station is indicated by the blue circle in the inset map. Bathymetry data were retrieved from General Bathymetric Chart of the Oceans (GEBCO Bathymetric Compilation Group (2024); https://www.gebco.net/data‐products‐gridded‐bathymetry‐data/gebco2024‐grid).

Compared with higher latitudes, whales in low latitude regions face greater exposure to potentially harmful human activities such as vessel collisions (Nisi et al. 2024), underwater noise (Shabangu et al. 2022), and fishing gear entanglement (Meÿer et al. 2011; Vermeulen et al. 2022). Marine protected areas (MPAs) are one management measure that can mitigate impacts of certain activities on whales, though only within their boundaries, by prohibiting such activities or imposing stricter controls on them than outside of their boundaries. In the marine area around continental South Africa, 41 MPAs exist following the declaration of 20 new MPAs in 2019 (South African National Biodiversity Institute 2018; Kirkman et al. 2021). One of these is the Child's Bank MPA that protects a unique submarine bank (Child's Bank) offshore of the west coast of South Africa, and its associated habitat and communities including cold‐water corals and associated fauna (Republic of South Africa 2019a; Figure 1a). Child's Bank is situated within the biologically productive Benguela Upwelling System (BUS) in the southeast Atlantic Ocean (Andrews and Hutchings 1980; Hutchings et al. 2009; Lamont et al. 2018). The BUS provides an essential habitat for many baleen whale species off South Africa and other countries on the western side of the southern African subregion that use this area for important biological activities such as breeding/calving, socialising, mating, feeding, overwintering and migration (Best 1996, 2001; Barendse and Best 2014; Barendse et al. 2011, 2013; Shabangu et al. 2019).

Whales off the west coast of South Africa used to be abundant enough to support a thriving whaling industry that operated from the Donkergat whaling station at Saldanha Bay (Figure 1a) from 1909 to 1967 during the modern whaling era (Best 2007). Subsequently, the area has been the focus of land‐ and boat‐based research, especially on humpback whales, which has provided evidence for increasing numbers, delayed migration, the presence of feeding supergroups, and other findings (e.g., Barendse et al. 2011, 2013; Findlay et al. 2017). This research has been largely observational, and until now, there has been very little long‐term acoustic research on baleen whales in this area (Becker et al. 2022; Darras et al. 2025). Baleen whales can be studied acoustically with high sound source identity levels and can be detected from a few tens to thousands of kilometres away (Letsheleha et al. 2022; Shabangu and Kowarski 2022; Shabangu et al. 2019; Shabangu, Andrew, et al. 2020; Shabangu, Munoz, et al. 2024). This is because they emit powerful, low‐frequency sounds that can be specific to a species, to a subspecies, or even to populations, as they may vary geographically (Filun and van Opzeeland 2023; Branch et al. 2025).

Initial attribution of detected sounds to a source is achieved via a variety of methods such as a combination of visual sighting and acoustic observations (Payne and McVay 1971; Parks et al. 2005; Webster et al. 2016; Allen et al. 2024), acoustic tagging (e.g., Risch et al. 2014) and knowledge of marine mammal spatio‐temporal distribution patterns (e.g., Allen et al. 2024). Most Northern Hemisphere studies on Bryde's whales suggest that this population produces pulses, burst tonals, downsweeps, growls and moans (Oleson et al. 2003; Heimlich et al. 2005) and most recently biotwangs (Allen et al. 2024). One study in South African waters found that Bryde's whales may produce single tones, multi‐tones, burst tonals and downsweeps (Faustmann et al. 2024). Southern right whales produce more than 10 call types (Webster et al. 2016), whereas humpback whales emit hierarchical songs in a thematic, sequential and predictable manner (Payne and McVay 1971; Cholewiak et al. 2013) and non‐song calls (e.g., Ross‐Marsh et al. 2022). Antarctic minke whales produce a variety of bioduck and downsweep calls (e.g., Risch et al. 2014; Dominello and Širović 2016; Shabangu, Findlay, and Stafford 2020; Shabangu, Munoz, et al. 2024; Filun and van Opzeeland 2023). All these sounds are produced for a wide range of purposes including communication, foraging, echolocation, swimming activities, reproductive advertisement, and antagonistic displays (Payne and McVay 1971; Parks et al. 2005, 2012; Risch et al. 2014; Helble et al. 2024). The range of sounds produced by baleen whales provides the basis for passive acoustic monitoring, not only to detect the occurrence of whale species but to monitor their vocalising patterns (e.g., diel variation) and behavioural response to underwater noise (Parks et al. 2011; Ross‐Marsh et al. 2021; Shabangu et al. 2019, 2021, 2022; Shabangu, Findlay, and Stafford 2020; Shabangu, Andrew, et al. 2020; Shabangu, Munoz, et al. 2024; Shabangu and Kowarski 2022).

One of the gazetted objectives of the Child's Bank MPA is to protect an adequate reference environment for research and monitoring (Republic of South Africa 2019a). Accordingly, this study used the MPA as its study site for passive acoustic monitoring of baleen whale populations. A specific objective of the study was to determine the acoustic occurrence of Bryde's whales, humpback whales, southern right whales, Antarctic minke whales and minke‐like whales relative to environmental conditions in the vicinity of the MPA in the southern African subregion. We also determined the diel vocalising patterns of these whales while measuring the acoustic characteristics of the previously undocumented southern African Bryde's whale offshore population call to aid other studies elsewhere in the Southern Hemisphere to identify the species.

## 2 Methods

### 2.1 Passive Acoustic Data Collection

Passive acoustic monitoring data were collected using two SoundTrap ST600 HF long‐term autonomous acoustic recorders (Ocean Instruments, New Zealand). These acoustic recorders were deployed on bottom‐mounted acoustic moorings (Figure 1b) in Child's Bank MPA off the west coast of South Africa (Figure 1a). The recorders were deployed 5 m from the bottom to reduce the impact of ocean current speed induced hydrophone flow noise but increase the probabilities of detecting marine mammal sounds (Shabangu et al. 2022). The Child's Bank MPA is approximately 125 km offshore from the coastal village of Hondeklip Bay and is approximately 1335 km^2^ in extent (Figure 1a). Protection is mainly focused on the benthic habitat and communities, so any form of bottom‐fishing including trawling is prohibited in the MPA (Republic of South Africa 2019b). For this reason, it was considered to be a suitable location for mooring deployments.

An acoustic mooring was deployed at a water depth of 281 m on 15 January 2024 (Table 1) with the intention for it to record until September 2024. However, it was trawled up 7 days after its deployment by a bottom‐fishing vessel that was in contravention of the MPA regulations (Shabangu et al. 2025). Following this disruption to data collection, a second acoustic mooring was deployed further northwards and closer to the centre of the MPA from May to October 2024 (Figure 1a; Table 1). The first acoustic mooring was 3.18 km from the nearest MPA boundary, whereas the second one was 12.8 km from the closest MPA boundary point (Figure 1a). Acoustic mooring set‐ups (Figure 1b) followed the same design described in Shabangu, Daniels, et al. (2024), Shabangu, Munoz, et al. (2024), Shabangu et al. (2025), and acoustic recorders were duty cycled to prolong the battery life of these instruments during the mooring deployment period (Table 1). A duty cycle of 14 min per hour was used for the January deployment to allow the instrument to collect data for 9 months, whereas a 45 min per hour duty cycle was used for the May–October deployment to maximise recordings during this relatively short deployment (i.e., 5 months).

**TABLE 1:** Locations of acoustic moorings and settings of autonomous acoustic recorders. ST is for SoundTrap.

Latitude (°S)	Longitude (°E)	Water depth (m)	ST depth (m)	Sample rate (kHz)	Sampling protocol (min h^−1^)	Duty cycle (%)	Hydrophone sensitivity (dB re 1 V/μPa)	Start recording date (dd/mm/yyyy)	End recording date (dd/mm/yyyy)
30.95	15.85	281	276	48	14	23	−176.3	15/01/2024	21/01/2024
30.83	15.84	192	187	48	45	75	−176.3	07/05/2024	11/10/2024

### 2.2 Acoustic Occurrence of Whales

**FIGURE 2:**
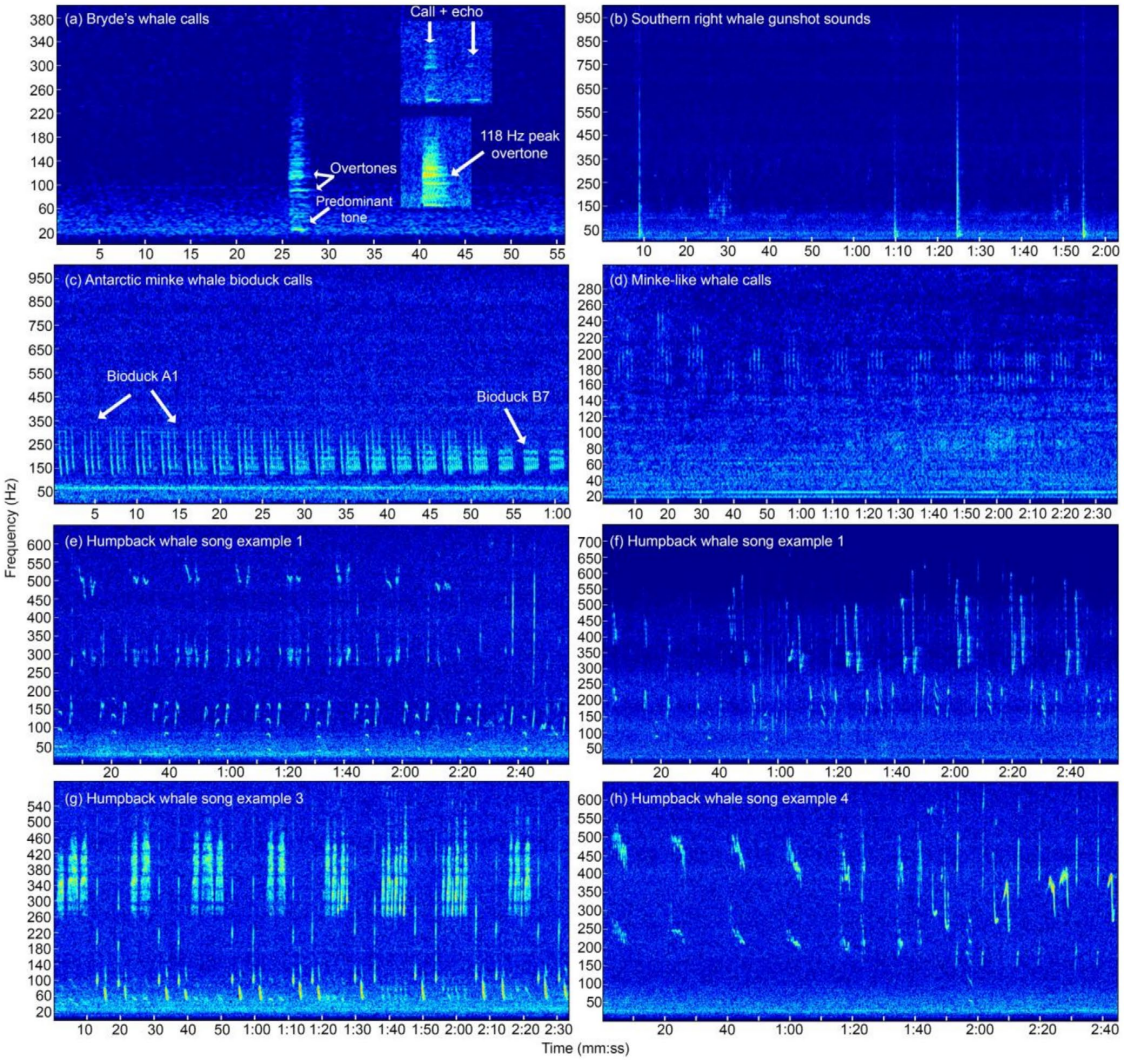
Spectrograms of (a) Bryde's whale burst tonal call BeSA1, (b) southern right whale gunshot sounds, (c) Antarctic minke whale bioduck A1 and B7 produced simultaneously by two whales, (d) minke‐like whale call, (e–h) examples of different humpback whale songs. Inset spectrograms in (a) are BeSA1 call with echo (Call+echo) and high‐quality BeSA1 call with a peak frequency of 118 Hz at the overtone. Different scales are used for the *y*‐axis of spectrograms. All spectrograms were produced using a Hann window, 90% overlap and DFT of 8192 samples. Window frame size for each spectrogram: (a) 0.775 s, (b) 0.330 s, (c) 0.230 s, (d) 0.838 s, (e) 0.677 s, (f) 0.724 s, (g) 0.724 s and (h) 0.677 s.

Given the sample rate of 48 kHz used to collect the acoustic data, we first decimated the acoustic data from 48 to 4 kHz using the ‘PAMmisc’ package (Sakai 2022) in R statistical software version 4.3.1 (R Core Team 2023). This file decimation improved the frequency resolution and the discrete Fourier transform (DFT) length of spectrograms when analyzing low frequency calls of baleen whales. To detect low frequency calls below 160 Hz, acoustic data were visually reviewed using spectrograms (Figure 2) in Raven Pro (K. Lisa Yang Center for Conservation Bioacoustics 2025) using a Hann window with a frame size of 1.28 s, 90% overlap and DFT size of 16,384 samples. For detection of low to medium frequency calls below 600 Hz, acoustic data were visually reviewed using a Hann window with a frame size of 0.724 s, 90% overlap and DFT size of 8192 samples. Sounds of interest were aurally reviewed to confirm or determine the sound source. Acoustic occurrence refers to the presence of one or more sounds within a sampling interval of 14 and 45 min for the January and May–October deployments, respectively. The use of different duty cycles between the two data sets is considered not to influence the results of this study since these sampling regimes have been found to yield comparable indices of whale acoustic presence (Thomisch et al. 2015). The occurrence of calls within these sampling intervals represented the hourly occurrence of whales per day.

Bryde's whale calls (Figure 2a) were identified and attributed to the species using comparison to spectrograms and characteristics (Table 2) of calls detected in the Northern Hemisphere (e.g., Oleson et al. 2003; Heimlich et al. 2005) and in consultation with one acoustic expert. Examples from Shabangu et al. (2021) were used to identify and classify southern right whale gunshot sounds (Figure 2b). Antarctic minke whale calls found in our data set (Figure 2c) were classified as bioduck A1 and B7 based on published literature from Antarctica (Dominello and Širović 2016; Shabangu, Findlay, and Stafford 2020; Filun and van Opzeeland 2023) and South African waters (Shabangu, Findlay, and Stafford 2020). Calls from an unknown source (Figure 2d) that resemble Antarctic minke whale bioduck calls were also detected, and these will be referred to hereafter as minke‐like whale calls. Humpback whale songs (Figure 2e–h) were identified using examples from Shabangu and Kowarski (2022) and Shabangu, Munoz, et al. (2024). Downsweep calls were attributed to humpback whales since these were produced in a typical hierarchical humpback whale song pattern comprising units, phrases and themes (Figure 2g,h), but not in a series as typically known for Bryde's whales (e.g., Faustmann et al. 2024). Additionally, these downsweep calls were different spectrally from the downsweeping (~106 to 22 Hz) Antarctic blue whale ( *B. musculus intermedia* ) D‐calls previously found on the west coast of South Africa (Shabangu et al. 2019). Likewise, upcalls that were detected with other humpback whale song units were attributed to this species.

**TABLE 2:** Summary of Bryde's whale call characteristics from previous studies. Values in brackets are ranges and — represents a case when a parameter was not measured. ICI is for inter‐call interval.

Call name	Location	Peak frequency (Hz)	Duration (s)	ICI (min)	References
Be3	Eastern Tropical Pacific	25.6 (24.4–26.91)	1.7 (1.0–4.0)	2.5 (0.45–8.65)	Oleson et al. (2003)
Low burst tonal	Eastern Tropical Pacific	29.8 (24.1–36.1)	2.1 (0.4–4.0)	5.8 (1.7–33.8)	Heimlich et al. (2005)
Burst tonal	Southeast Atlantic Ocean	41.8 (35.2–74)	0.34 (0.13–2.5)	—	Faustmann et al. (2024)

### 2.3 Diel Vocalising Behaviour

Diel acoustic occurrence of call and song types was used to determine the diel vocalising patterns of each whale species over different daylight regimes. Data from January were not used for this analysis due to the duration of the recording period being too short to infer any meaningful long‐term biological information. Daylight regimes for the May–October data set were obtained from the acoustic mooring position using the ‘suncalc’ package (Thieurmel and Elmarhraoui 2019) in R. We defined the nautical daylight regimes (sunrise, sunset and nautical twilight) using the definitions provided in Shabangu, Munoz, et al. (2024). Austral seasons were used to construe the data: December–February (summer), March–May (autumn), June–August (winter) and September–November (spring).

### 2.4 Call Characteristics Measurements

Characteristics of the previously undescribed southern African Bryde's whale call and minke‐like whale call were measured in Raven Pro. Measured call parameters were minimum frequency, peak frequency, maximum frequency, duration, inter‐call interval (ICI), inter‐pulse interval (IPI) and inter‐series interval (ISI). These parameters were determined from good‐quality calls with a minimum signal‐to‐noise ratio of 6 dB. Minimum frequency of a call represented the lowest frequency limit, maximum frequency of a call represented the highest frequency limit, and peak frequency indicated the frequency at which peak power occurs within a call. Duration was calculated as the time difference between the start and end of a call that represented the 100% duration of calls. On the other hand, ICI was determined for Bryde's whale calls only as the time difference between the start of one call and the start of the next, and this was determined from calls deemed to be produced by a single whale and not by multiple whales. The presence of one or two non‐overlapping, regularly spaced calls within a 180 s spectrogram window was taken to represent instances when one whale was vocalising, whereas three or more calls within a 180 s spectrogram window were taken to represent the presence of multiple whales calling as per published ICI ranges in Table 2. Furthermore, calls produced by multiple whales had irregular and non‐rhythmic patterns. For minke‐like whale calls, ISI and IPI were measured using a method described in Shabangu, Munoz, et al. (2024). Statistics of call characteristics were determined using the ‘pastecs’ package (Grosjean and Ibanez 2018) in R.

### 2.5 Data on Environmental Conditions

To establish relationships between the environment and whales, daily chlorophyll‐a (mg m^−3^), daily sea surface temperature (SST; °C), daily sea surface height (SSH; m) and hourly eastward and northward wind speed (m s^−1^) data for the May–October 2024 period were retrieved from Copernicus Marine Environment Monitoring Service (CMEMS) satellite‐derived and ocean reanalysis products (Table 3). No environmental data were accessed for the January 2024 period because this recording period was too short to be able to meaningfully relate whale occurrence to environmental variables. Chlorophyll‐a was used as an indication of primary production and phytoplankton biomass; SST was used as a measure of physical processes that affect primary productivity; SSH was used as a proxy of ocean circulation; and wind speed was used as an indication of the ocean state.

**TABLE 3:** Links to environmental data sources and descriptions together with the spatial resolution of each variable.

Variable	Data description and access link	Spatial resolution
Chlorophyll‐a	https://data.marine.copernicus.eu/product/OCEANCOLOUR_GLO_BGC_L4_MY_009_104	4 × 4 km
SST	https://data.marine.copernicus.eu/product/SST_GLO_SST_L4_NRT_OBSERVATIONS_010_001	4 × 4 km
SSH	https://data.marine.copernicus.eu/product/SEALEVEL_GLO_PHY_L4_NRT_008_046	14 × 14 km
Wind speed	https://data.marine.copernicus.eu/product/WIND_GLO_PHY_L4_MY_012_006	4 × 4 km

The maximum detection ranges of baleen whale sounds were assumed as follows: 30 km for southern right whale gunshot sounds (Shabangu et al. 2021), 10 km assumed for Bryde's whale calls (Širović et al. 2014), 18 km for humpback whale songs (Shabangu and Kowarski 2022), and 27 km for Antarctic minke whale bioduck calls (Filun et al. 2020). We acknowledge that the above maximum detection ranges will vary between locations due to changes in environmental conditions, bathymetry and noise levels (e.g., Letsheleha et al. 2022; Shabangu et al. 2019, 2021, 2022); nonetheless, these provide rough estimates of the whale call maximum propagation ranges to be expected in this region. Environmental data for all species were then extracted and averaged for an area up to 30 km from the acoustic mooring location, which corresponds to the southern right whales' maximum detection range as provided above. Eastward and northward wind speeds were converted to absolute wind speed using the formula provided in Shabangu et al. (2022), Shabangu, Munoz, et al. (2024) and Shabangu, Daniels, et al. (2024) and the hourly values were then averaged to a daily scale to match the temporal scale of other environmental variables. Given that all environmental data were now at a daily scale while whale acoustic occurrence was at an hourly scale, the influence of environmental conditions on acoustic occurrence were interpreted on a daily basis.

### 2.6 Statistical Data Analysis

To determine the influence of predictor variables (month, hour, chlorophyll‐a, SST, SSH and wind speed) on the May–October daily acoustic occurrence of the four baleen whale species studied here, excluding the minke‐like whales, we utilised random forest (RF) models (Breiman 2001). The minke‐like whales were excluded from this analysis because their low acoustic occurrence (< 20) would have been insufficient for the RF model to produce accurate prediction results (e.g., Luan et al. 2020). All other species had acoustic occurrence sample sizes greater than 69 and were retained for use in RF models as they were likely to produce accurate RF model predictions. January was excluded from this analysis due to its short recording duration. The RF models were preferred for use in this study given their superior performance at describing marine mammal occurrence over some of the commonly used models such as generalised boosted regression trees models and generalised additive models (Shabangu et al. 2017, 2019). Distinctive and powerful capabilities of RF models as a machine learning method include that they have high prediction accuracies and non‐parametric inferential properties while including implicit interaction between predictor variables (Breiman 2001; Hastie et al. 2009; Ziegler and König 2014). The RF models were fitted using the ‘randomForest’ R package (Liaw and Wiener 2002) which was implemented through the ‘ranger’ package (Wright and Ziegler 2017) to fit RF models faster.

Prior to fitting the RF models, we tested for multicollinearity to ensure that no correlated variables were used in RF models, which improved the accuracy of RF models. Multicollinearity between predictor variables was estimated using generalised variance inflation factors (GVIFs) for continuous and categorical variables with just two levels, whereas the adjusted generalised standard error inflation factors (aGSIFs) were more suitable for categorical variables with more than two levels because it adjusts for the number of levels, allowing comparability with the other predictors (Fox and Monette 1992). GVIF and aGSIFs were determined through the ‘car’ R package (Fox and Weisberg 2019). A GVIF around one indicated no multicollinearity, while GVIF values ≥ 5 or > 10 indicated strong multicollinearity (O'Brien 2007). aGSIF values above 2.2 (which is the square root of GVIF threshold of 5) or 3.2 (square root of alternative GVIF threshold of 10) were indicative of strong multicollinearity. Strong multicollinearity was found between month, SSH and SST (Table 4), and SSH (*r* = 0.85) and SST (*r* = −0.85) were highly correlated to month. As a result, month was removed as a predictor variable, and observed GVIFs dropped to a maximum of 1.91 (Table 4), implying no to weak multicollinearity between predictor variables (O'Brien 2007).

**TABLE 4:** Multicollinearity results for predictor variables used in this study. Chlorophyll‐a was used as the reference variable in linear regression models. df is for degree of freedom. GVIF and aGSIF values in red and boldfaced represent strong multicollinearity. The boldfaced light blue value represents moderate multicollinearity.

Variables	df	With multicollinearity		Without multicollinearity	
		GVIF	aGSIF	GVIF	aGSIF
Hour	23	1.00	1.00	1.00	1.00
Month	5	**50.14**	1.48	—	—
SST	1	**15.10**	**3.89**	1.91	1.38
SSH	1	**6.36**	**2.52**	1.88	1.37
Wind speed	1	1.17	1.08	1.02	1.01

**FIGURE 3:**
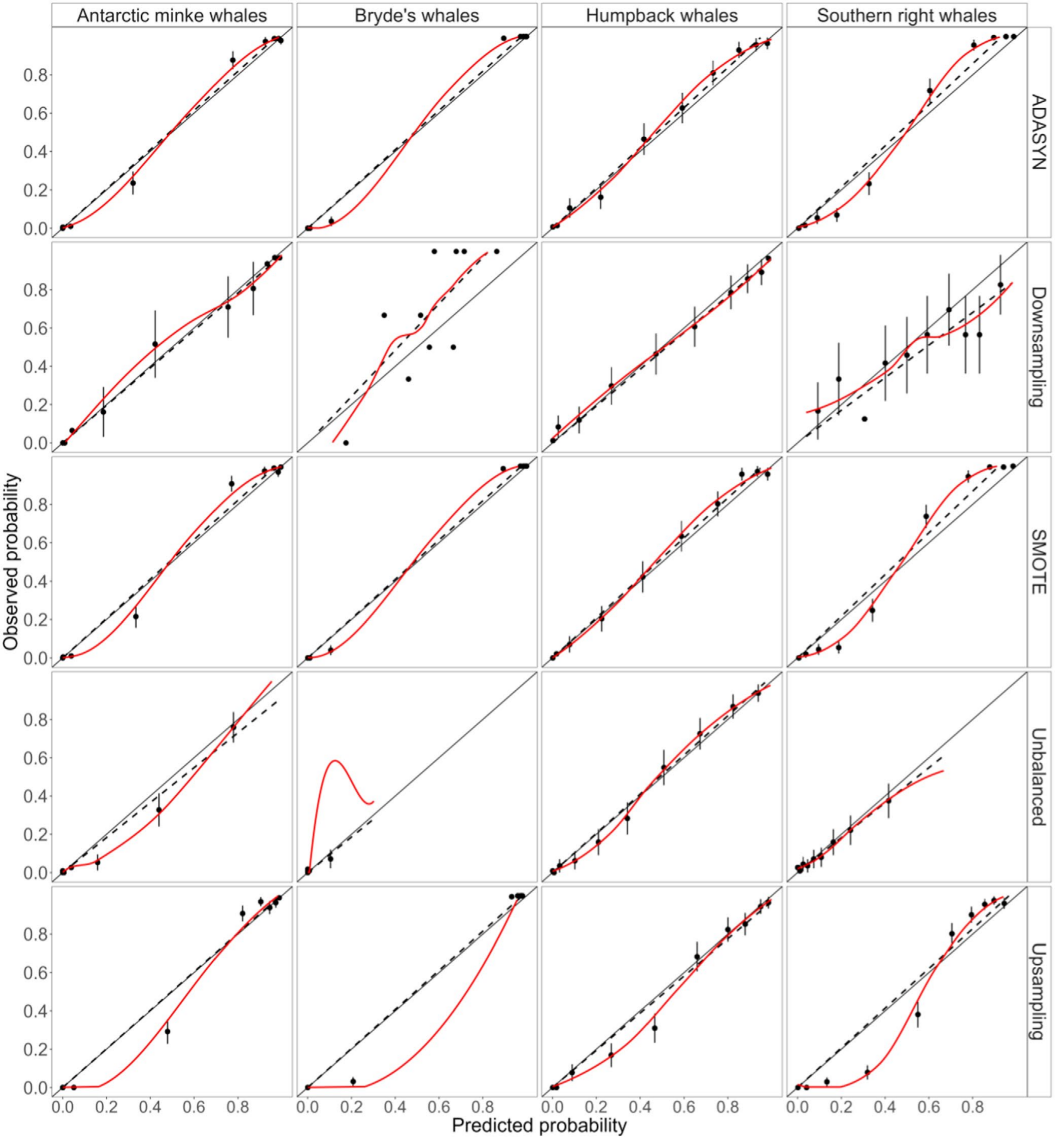
Calibration plots of different sample balancing methods used to visually evaluate the predictive performance of random forest (RF) models on tuned data for each baleen whale acoustic occurrence. Dashed lines represent the ideally calibrated prediction (where the area below the dashed line indicates cases when RF models' predictions were too high while the area above the dashed line indicates cases when the RF model's predictions were too low), closed circles represent the binned actual (true) observed data ± confidence intervals of the bin (whiskers), red lines represent the RF model's predicted probabilities based on different sample balancing methods, and the continuous lines represent equal split between observed and predicted probabilities.

Due to the large difference in the amount of time between positive and negative whale acoustic occurrence (i.e., presence vs. absence), four sample balancing procedures were explored: Synthetic Minority Over‐sampling Technique (SMOTE; Chawla et al. 2002), ADAptive SYNthetic (ADASYN; He et al. 2008), downsampling and upsampling (Nallamuthu 2020). SMOTE and ADASYN sample balancing methods had predicted probabilities that were more comparable to each other than to the other methods (Figure 3). As such, similar results would have been produced between the two methods (Shabangu, Munoz, et al. 2024), and we arbitrarily chose SMOTE for this study.

RF models were tested using 30% of the balanced data, and the remaining 70% of the data were used for training (tuning the model). The training data set was further set up for fivefold cross‐validation, which was used in succession for tuning the model. Predictive performances of all the RF models were then assessed on the test data set, which was not used when tuning the models. For these classification RF models, the best tuning parameters were chosen as sets that maximised the area under the curve of the receiver operating characteristics. All feature (i.e., sets of predictors/covariates in the model) importance values were scaled to the maximum for each model and presented as percentages. Statistical significance of the importance of each variable was tested using the permutation method (Altmann et al. 2010), which enabled smooth interpretation of RF model results. Variables of statistical significance were more informative predictors than variables that were non‐significant; though the latter were still ecologically important as predictors.

## 3 Results

### 3.1 Passive Acoustic Monitoring Effort

Over the acoustic mooring deployment periods, a total of 165 days and 2853 h were sampled by our acoustic recorders (Table 5). From the January data set, 31 h were recorded over 7 days, and 2 822 h were recorded over 158 days (5 months) from the May–October deployment (Table 5). Sampled months over this study period fully or partially represented different seasons of the year: summer (January), autumn (May), winter (June, July and August) and spring (October).

**TABLE 5:** Number of hours recorded and of days with acoustic recordings per month.

Month	No. of hours recorded	No. of days with recordings
January	31	7
May	441	25
June	540	30
July	558	31
August	558	31
September	540	30
October	185	11
Total	2853	165

### 3.2 Bryde's and Minke‐Like Whale Call Properties

In total, 83 and 272 Bryde's whale burst tonal calls (Figure 2a) were identified in the January and May–October data sets, respectively. These Bryde's whale burst tonal calls had predominant tones and overtones and general properties summarised in Table 6. Given that these are the first Bryde's whale call observations for the offshore population in the southern African subregion, we are terming this call BeSA1, short for *Balaenoptera edeni* southern Africa 1. Minke‐like whale calls had five pulses per series with properties detailed in Table 6 and were produced in a song form.

**TABLE 6:** Average ± SD (range, sample size [*n*]) of properties of Bryde's whale burst tonal calls (BeSA1) and minke‐like whale calls.

Call type	Minimum frequency (Hz)	Maximum frequency (Hz)	Peak frequency (Hz)	Duration (s)	ICI (minutes)	IPI (s)	ISI (s)
BeSA1	19.16 ± 1.57 (16.09–26.55, *n* = 51)	260.07 ± 88.87 (156.06–548.61, *n* = 51)	30.43 ± 19.43 (23.44–118.16, *n* = 51)	2.32 ± 0.39 (1.75–3.17, *n* = 51)	4.48 ± 2.07 (1.22–15.14, *n* = 147)	—	—
Minke‐like	143.68 ± 12.54 (111.10–170, *n* = 49)	199.64 ± 15.07 (166.30–245.50, *n* = 49)	173.59 ± 19.21 (142.58–234.37, *n* = 49)	0.76 ± 0.12 (0.52–0.97, *n* = 49)	—	1.32 ± 0.17 (1.03–1.93, *n* = 36)	11.48 ± 1.88 (7.92–14.81, *n* = 11)

*Note:* ‘—’ indicates that acoustic property was not measured.

### 3.3 Whale Acoustic Occurrence

**FIGURE 4:**
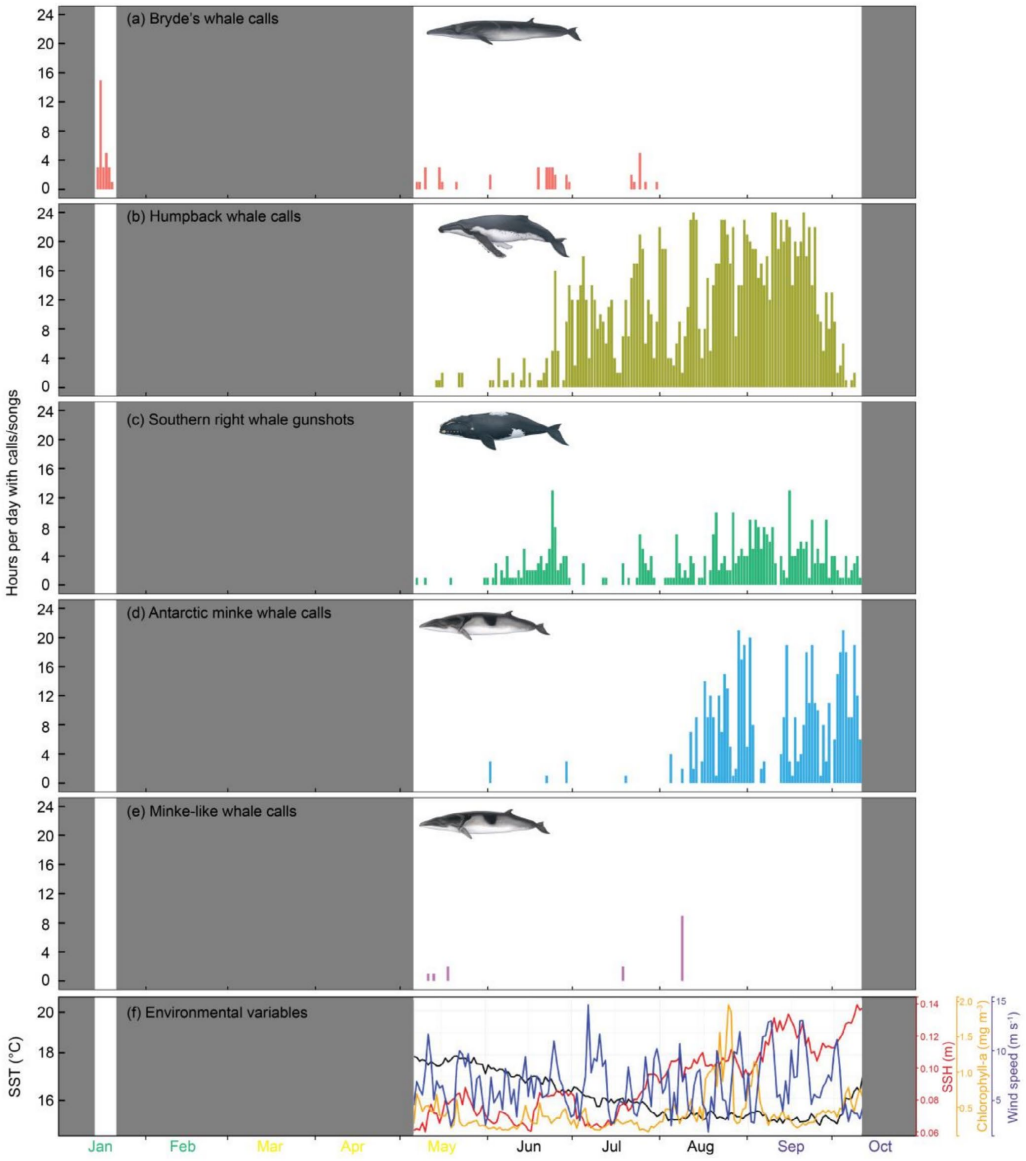
(a–e) Number of hours per day with whale calls/songs and (f) daily trends in environmental conditions at the Child's Bank MPA off the west coast of South Africa. Seasons are indicated by the font colour of month, where green is for summer, orange is for autumn, black is for winter and purple is for spring. Grey shadings represent periods without passive acoustic monitoring effort.

Bryde's whale calls were detected in January and intermittently in May through July but not in August through October, and January had the highest occurrence (Figure 4a). Humpback whale songs and southern right whale gunshot sounds were not detected in January but were detected from May through October, with the highest occurrence of sounds in September and small modes in other months (Figure 4b,c). Antarctic minke whale calls were not found in January and May but were detected in June through October (Figure 4d). The highest number of hours with Antarctic minke whale calls was in August, September and October. Antarctic minke whale bioduck A1 and B7 (Figure 2d) were detected in equal proportions from the May–October data set, with bioduck A1 being prominent in the first few months (May–July) and then bioduck B7 dominating during the last few months (August–October). Minke‐like whale calls were only detected in May, July and August, with the highest number of hours with calls occurring in August (Figure 4e). No sounds of toothed whales such as sperm whales were detected from these acoustic recordings. Daily trends of environmental conditions over months are shown in Figure 4f, which highlights strong changes in SSH and SST with month, as emphasised by correlation coefficients calculated in Section 2.6.

### 3.4 Detected Diel Pattern of Vocalisation

**FIGURE 5:**
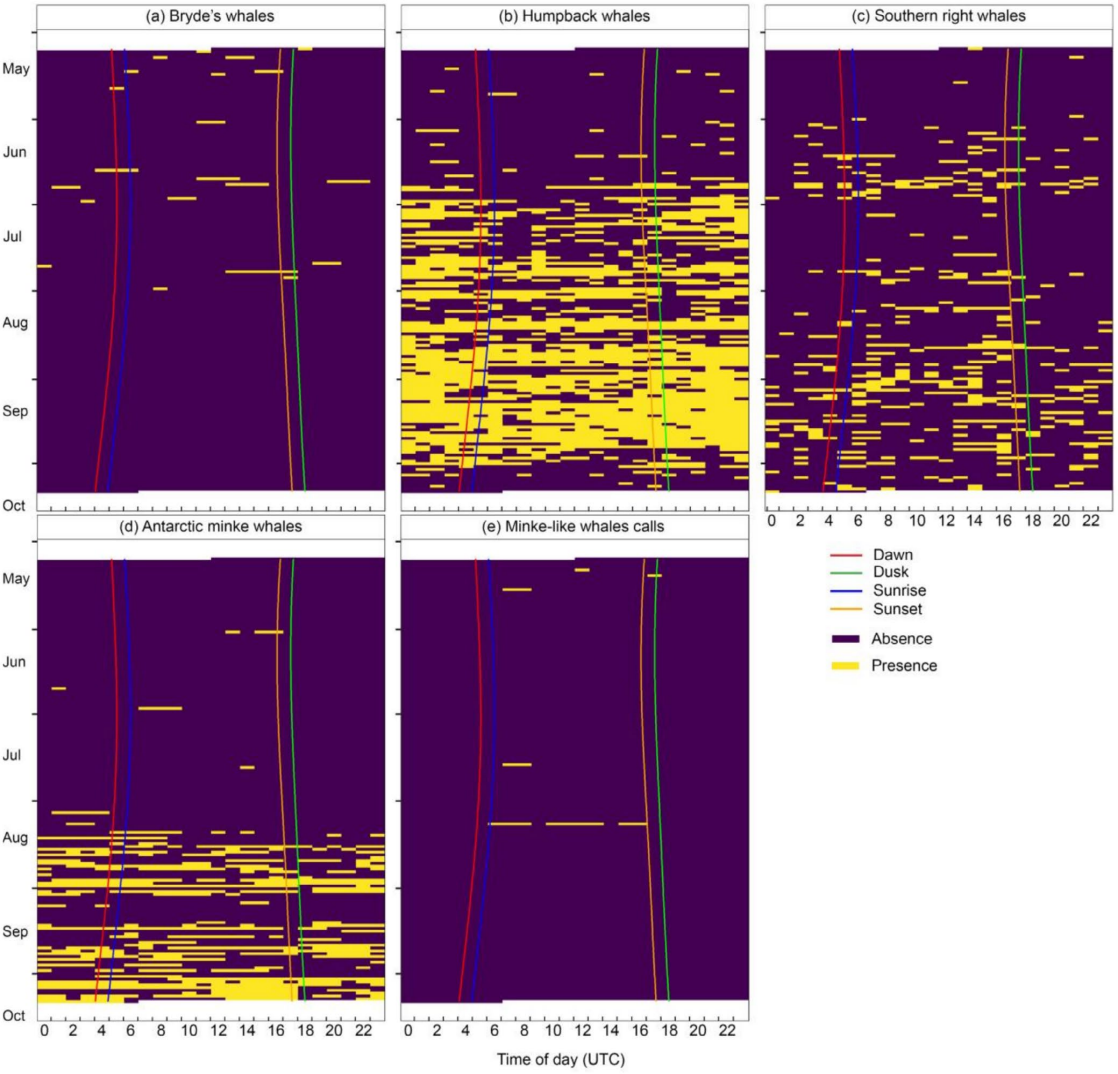
Diel acoustic occurrence of whales based on presence and absence of sounds from May to October. Time is in Coordinated Universal Time (UTC) zone, and daylight regime description is provided in key. White shadings represent periods without passive acoustic monitoring effort.

Bryde's whales, southern right whales and minke‐like whales were observed to be more vocally active during the day (Figure 5a,c,e). Humpback whale songs were more present at night (Figure 5b). Antarctic minke whale bioduck calls were detected during the day in June and July, but were present throughout the day in August to October (Figure 5d). Minke‐like whale calls were detected purely during daytime and not at night, with dusk call detection as the closest time to nighttime (Figure 5e).

### 3.5 Whale Acoustic Occurrence Predictors

**FIGURE 6:**
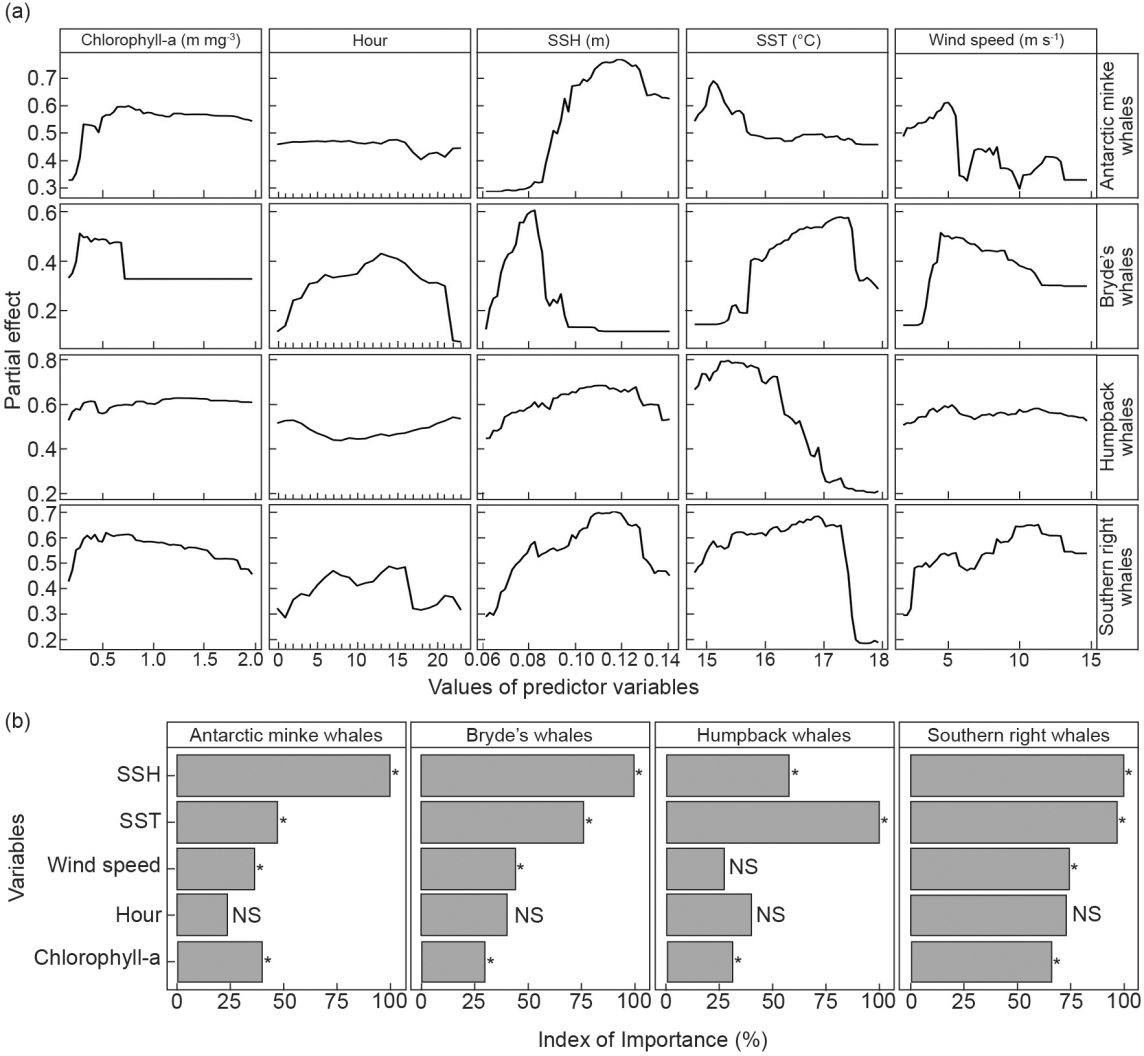
Random forest (RF) models (a) influence and (b) importance of predictor variables on the acoustic occurrence of baleen whales for the May–October period determined using the Synthetic Minority Over‐sampling Technique (SMOTE) sample balancing method. NS indicates statistically non‐significant (*p* > 0.05) importance and an asterisk (*) indicates statistically significant (*p* < 0.05) importance.

The acoustic occurrence probability of all baleen whale species increased with chlorophyll‐a to or before 0.3 mg m^−3^ and decreased or flattened after this level (Figure 6a). Hours after midnight to late afternoon (16:00) had the highest influence on Antarctic minke whale acoustic occurrence, while daytime hours had the highest influence on Bryde's and southern right whale acoustic occurrence (Figure 6a). On the other hand, nighttime hours had the highest influence on humpback whale acoustic occurrence. Acoustic occurrence of Antarctic minke increased with SSH, and Bryde's whale acoustic occurrence increased with SSH from 0.08 to 0.12 m and decreased thereafter (Figure 6a). Humpback and southern right whale acoustic occurrence increased at fluctuating rates with SSH to 0.12 m and then decreased after that. Bryde's whale acoustic occurrence increased with SSH to 0.08 m and sharply decreased thereafter before plateauing. Acoustic occurrence of Antarctic minke whales increased with SST from 14.8°C to 15.2°C and then gradually decreased thereafter (Figure 6a). Humpback whale acoustic occurrence increased with SST from 14.8°C to 15.9°C and decreased subsequently. Acoustic occurrence of Bryde's whales increased with SST from 15.2°C to 17.4°C and decreased sharply after this level (Figure 6a). Southern right whale acoustic occurrence increased with SST to 17.3°C and dropped afterwards. Antarctic minke and Bryde's whale acoustic occurrence first increased with wind speed to 5 m s^−1^ and then decreased at different rates (Figure 6a). Humpback whale acoustic occurrence fluctuated around the same level when wind speed increased, while southern right whale acoustic occurrence increased with wind speed at fluctuating rates.

SSH was the most important predictor of Antarctic minke and Bryde's whale acoustic occurrence; SST was the most important predictor of humpback whale occurrence; and SSH and SST were the most important predictors of southern right whales (Figure 6b). SST, wind speed and chlorophyll‐a were the moderately important predictors of Antarctic minke whale acoustic occurrence, whereas SST was the moderately important predictor of Bryde's whale acoustic occurrence. SSH and hour were the moderately important predictors of humpback whale acoustic occurrence, and hour and wind speed were the moderately important predictors of southern right whales (Figure 6b). Hour was the least important predictor of Antarctic minke whale acoustic occurrence; hour, wind speed and chlorophyll‐a were the least important predictors of Bryde's whale acoustic occurrence; wind speed and chlorophyll‐a were the least important predictors of humpback whale acoustic occurrence; wind speed and chlorophyll‐a were the least important predictors of southern right whales. Depending on the whale species, some predictors were significantly important, whereas some were not (Figure 6b).

## 4 Discussion

This study provides novel information about the acoustic occurrence of baleen whales for a part of the southern African subregion that was previously unsampled using passive acoustic monitoring technology. We show that multiple baleen whale species are acoustically present in or around Child's Bank MPA, with some temporal overlap in acoustic occurrence. Importantly, the acoustic occurrence of these whales is strongly driven by environmental conditions. We provide characteristics of the southern African Bryde's whale offshore population to equip future passive acoustic studies with the necessary knowledge to study this species, which is one of the less studied baleen whale species in the world. This study highlights the potential role of an MPA that is focused on benthic protection for the conservation of baleen whales by reducing potential threats within its boundaries.

### 4.1 Bryde's Whale Call Characteristics

Bryde's whales that occur in the southern African subregion are classified into two populations, inshore and offshore, based on their distribution (Best 1977, 2001, 2007) and genetics (Penry et al. 2018). Given that the offshore location of our recording site (Child's Bank) overlaps only with the distribution of the southern African Bryde's whale offshore population (Best 1996, 2001, 2007), our detected Bryde's whale calls can confidently be attributed to this population. The general spectral structure and characteristics of the BeSA1 call (Table 6) reported in this study resemble those of Be3 and low burst tonal calls (Table 4) from Bryde's whales recorded offshore in the Northern Hemisphere (Oleson et al. 2003; Heimlich et al. 2005). The BeSA1 minimum frequency of 16 Hz measured in this study is slightly higher than the lowest minimum frequency of 10 Hz in Helble et al. (2024). Similarly, the BeSA1 maximum frequency is significantly higher than the Be3 maximum frequency of the 200 Hz maximum mentioned in Helble et al. (2024). The high maximum frequency of BeSA1 calls indicates that vocalising whales were close to the acoustic recorder location to enable detection of higher frequency harmonics of these calls. The average peak frequency of BeSA1 (30.43 Hz) falls within the range of the peak frequency of the Northern Hemisphere Bryde's whale calls of 24.1–36.1 Hz (Oleson et al. 2003; Heimlich et al. 2005). Some of the observed calls had echoes (Figure 2a), similar to those observed in Helble et al. (2024), indicating that vocalising animals were very close to the acoustic recorder location to allow multipath reception given that echo strength decreases with range from the source (e.g., Shabangu et al. 2021). Spectral characteristics of BeSA1 look similar to those of Bryde's whale associated calls recorded in New Zealand waters (Kibblewhite et al. 1967; McDonald 2006), although the New Zealand calls are much longer (~5 s) in duration than the calls reported here. The BeSA1 spectral characteristics are also similar to burst tonal type, TM1, from Bryde's whales recorded in Brazil (Figueiredo and Simão 2014).

The global similarity of these calls between allopatric populations suggests that these calls might be innate as postulated for humpback whales (Fournet et al. 2018). The 118 Hz overtone peak frequency measured in this study for some high signal‐to‐noise ratio calls suggests that the overtones of Bryde's whale calls can sometimes have higher energy than the predominant tone, which was not reported previously. However, the BeSA1 characteristics (peak frequency and duration) are slightly different from burst tonal calls of the southern African Bryde's whale inshore population (peak frequency: 42 Hz and duration: 0.34 s) detailed in Faustmann et al. (2024), suggesting that these two populations can be differentiated acoustically. The BeSA1 acoustic characteristics provided here represent a significant contribution to the understanding and global research objectives for this species that is considered to be one of the lesser‐known baleen species due to its cryptic and solitary nature (Constantine et al. 2018).

### 4.2 Acoustic Occurrence Pattern

Monthly acoustic occurrence established for the species in this study advances our species knowledge and establishes important baseline information that can be useful for conservation and management strategies throughout the southern African subregion. Given the maximum detection range of these baleen whale calls (provided in Section 2.5), the presence of Bryde's whale call echoes and high‐quality calls from other whale species, it is probable that the sources of all sound detections were within the MPA or nearby it. The high Bryde's whale acoustic occurrence in January in this study, from the data set collected with the shortest duty cycle, confirms the effectiveness of our duty cycles in producing comparable indices of acoustic occurrence. It should be noted that passive acoustic monitoring only detected vocally active whales and missed non‐vocalising whales.

The fact that Bryde's whales were acoustically present in or near Child's Bank in January and May–July suggests that they may have been in the area for an extended period of time (i.e., 7 months) since they were detected after almost 4 months of recording disruption. They could potentially have been feeding on small pelagic fish (sardine ( *Sardinops sagax* ) and anchovy ( *Engraulis capensis* )) and euphausiids (Best 1977, 2001, 2007) found on and around Child's Bank. The absence of their calls between August and October could reflect that these whales migrated further offshore or out of the area to other nearby habitats (e.g., Best 2001) not covered by our acoustic recorder, possibly to track shifts in prey availability, or alternatively were just silent in these months due to changes in behavioural state.

Southern African Bryde's whales are currently categorised as Vulnerable by The Red List of Mammals of South Africa, Lesotho and Swaziland (Penry et al. 2016) and thus need special conservation efforts to avoid further population decline. The fishing regulations of the Child's Bank MPA affords these and other whales a measure of protection in this relatively shallow offshore environment, especially from entanglement in fishing gear, by prohibiting bottom‐fishing, including methods that require surface marker buoys to be anchored to the seafloor via rope that is often under tension (e.g., demersal longlining or traplines). Baleen whales, especially Bryde's whales because of their ‘high‐speed chases near the seafloor to catch their prey’ mode of feeding (Segre et al. 2022), are highly susceptible to entanglement in such gear (e.g., Meÿer et al. 2011; Vermeulen et al. 2024). The only modes of fishing allowed in the MPA are tuna pole fishing and pelagic longline fishing (Republic of South Africa 2019b). The latter, which uses gear that is designed to be suspended in the water column, poses much less entanglement risk to baleen whales than to toothed whales, which are attracted to the bait or catch (Fader et al. 2021). Elsewhere, however, baleen whales such as Bryde's whales and humpback whales have been shown to also be vulnerable to entanglement in pelagic longline gear (Forney 2010; Forney et al. 2011) and they remain at risk of this in the MPA. Ship strikes and underwater noise from ships are additional pressures that the MPA does not provide protection against, since there are no restrictions on vessel traffic through the MPA (Republic of South Africa 2019b), which is in the path of a major shipping lane (e.g., figure 1b of Nisi et al. 2024). Shipping traffic was previously identified by Purdon, Shabangu, Pienaar, et al. (2020) as one of the key stressors to cetaceans within the South African exclusive economic zone.

Acoustic occurrence of humpback whale songs from May through October found in this study is slightly earlier than June through November occurrence that was observed further offshore by Shabangu and Kowarski (2022), but similar to the acoustic occurrence of this species around the sub‐Antarctic Prince Edward Islands (Shabangu, Munoz, et al. 2024) and off Namibia (Thomisch et al. 2019). The peak month for humpback whale singing in this study, September, is similar to the pattern recorded by Shabangu and Kowarski (2022) for one of their study sites off the west coast of South Africa in 2016, but is earlier than the October peak that they recorded for all their other study sites (*n* = 3) in this region between 2014 and 2017. This difference in acoustic occurrence might be related to temporal migration patterns, habitat usage, environmental conditions, and vocalising behavioural variations between these areas (Thomisch et al. 2019; Shabangu and Kowarski 2022; Shabangu, Munoz, et al. 2024). Despite that the singing peak at Child's Bank was earlier than at other locations, it still falls within the breeding season of this breeding stock (Best 2007).

The intermittent detection of southern right whale gunshot sounds from May through October corresponds to inshore sightings of this species at Saldanha Bay on the west coast of South Africa (Barendse and Best 2014). It also overlaps with but does not exactly match the acoustic occurrence of southern right whale recorded by Shabangu et al. (2021) around the deep (855 m) offshore area off Cape Point on the west coast of South Africa, which was from August to December. Acoustic occurrence from the relatively shallow Child's Bank station (190 m water depth) is also higher than that recorded by Shabangu et al. (2021) off Cape Point, which suggests that the Child's Bank shallower environment, which is potentially associated with greater productivity, might be more appealing to this species (e.g., Purdon, Shabangu, et al. 2020). While the physical oceanographic processes and ecosystem responses that govern the local Child's Bank environment are yet to be characterised, it is likely that the waters above and around the Bank will be productive due to its location within the BUS. Cold, nutrient‐rich waters that are upwelled along the South African coast warm as they are advected offshore, allowing for the development of phytoplankton communities and subsequent ecosystem responses, which are expected to peak in the mid‐shelf region where the Bank is located (Hutchings et al. 2009; Lamont et al. 2015). In comparison, off Cape Point, the productive waters of the BUS are constrained to the much narrower shelf region, and thus the waters in the open ocean region are much warmer and less productive (Lamont et al. 2018). This difference in productivity likely accounts for the higher presence at Child's Bank compared to the findings of Shabangu et al. (2021) and is in agreement with previous suggestions that shallower environments with greater productivity may be more appealing to this species (e.g., Purdon, Shabangu, Yemane, et al. 2020).

Antarctic minke whale bioduck calls were detected from June through October, preceding their recorded acoustic occurrence off the west coast of South Africa (August through December) by 2 months (Shabangu, Findlay, and Stafford 2020). Off Namibia, Thomisch et al. (2019) detected Antarctic minke whale bioduck calls at various recording locations from June through August, which is closer to the timing observed in this study. We found Antarctic minke whale acoustic occurrence with three peaks in August, September and October, whereas the acoustic occurrence peak in Shabangu, Findlay, and Stafford (2020) over 2 years off the west coast of South Africa was in September/October. On the east coast of the southern African subregion in the Mozambique Channel, Antarctic minke whale calls were detected from June through December (Dréo et al. 2025), which somewhat corresponds to June–October in terms of commencement of acoustic occurrence. In Antarctica, Antarctic minke whale bioduck calls were detected from April through January (Filun et al. 2020; Shabangu, Findlay, and Stafford 2020). Remarkably, Child's Bank has a higher Antarctic minke whale acoustic occurrence than the sub‐Antarctic Prince Edward Islands (Shabangu, Munoz, et al. 2024), potentially highlighting the waters off the west coast of South Africa as a favourable habitat for this species. Alternatively, it may be a case of these whales vocalising more frequently at Child's Bank in the absence of their main predator, killer whales ( *Orcinus orca* ), calls of which were not detected in this study. In contrast, killer whales were acoustically present year‐round at the Prince Edward Islands (Shabangu, Daniels, et al. 2024). Short‐term migration movements and behavioural changes between these locations may influence the observed differences in acoustic occurrence between them. Of note is that the seasonal peak in Antarctic minke whale calling period in this study corresponds with their breeding season (Best 2007). Similarly, the Antarctic minke whale seasonal call type switch from bioduck A3 to bioduck B7 might indicate acoustic behavioural change from the beginning (June) of the breeding season to the peak (September/October) of the breeding season. The minke‐like whale calls are likely produced by a different species given their temporal segregation with Antarctic minke whale calls in most months (Figure 4) and their acoustic characteristics that differ from those previously reported for Antarctic minke whales (e.g., Risch et al. 2014; Dominello and Širović 2016; Shabangu, Findlay, and Stafford 2020; Shabangu, Munoz, et al. 2024; Filun and van Opzeeland 2023).

The winter (June through August) and early spring (September) peak acoustic occurrence of these baleen whales on and around Child's Bank suggests that these whales could be overwintering and/or feeding in the waters off the west coast of South Africa or at least use this area as their migration route to and from their breeding grounds (Best 1996, 2011; Best and Allison 2010; Best et al. 1995). Here, they may benefit from the ecosystem protection provided by the Child's Bank MPA, including exclusion of several modes of fishing and oil and gas‐related activities. Overall, the occurrence of these baleen whales as determined through our passive acoustic monitoring effort underlines the potential usefulness of this method to study some of the rarely sighted and lesser‐known species in the Southern Hemisphere. The absence of toothed whale calls previously detected year‐round in the offshore region of the west coast of South Africa (Shabangu and Andrew 2020) motivates further passive acoustic monitoring research in South African waters and other areas in the southern African subregion.

### 4.3 Diel Vocalising Pattern

Daytime vocal activity of Bryde's whales observed here is different from the dusk and nighttime observations in the Gulf of Mexico (Širović et al. 2014), highlighting special adaptation to the local environment and possibly diel vertical migration of their prey, small pelagic fish (e.g., Zwolinski et al. 2007). This would imply that Bryde's whales vocalised more during the day when foraging on prey at depth and vocalise less at night while resting, similar to a behaviour observed in New Zealand (Izadi et al. 2018). This is the first report of Bryde's whale diel vocalising patterns in the southern African subregion and the whole of the Southern Hemisphere, providing important behavioural insights for understanding the ecology of this species. Changes in diel vocalising patterns of southern right whales observed from Child's Bank may also be explained by changes in behavioural sound production contexts related to prey diurnal vertical migration, and mating, resting, or swimming activities (Parks et al. 2005, 2012). Shabangu et al. (2021) found the southern right whale diel vocalising pattern to vary depending on the sampling site and season—the pattern at this location matched the high daytime vocalisation patterns in summer and winter from the station at 855 m water depth of Shabangu et al. (2021). Humpback whale vocal activity was highest at nighttime to maintain communication in the dark. This observation conforms to previous humpback whale diel vocalising patterns based on recordings off the west coast of South Africa (Ross‐Marsh et al. 2021; Shabangu and Kowarski 2022), Antarctica (Shabangu and Kowarski 2022) and the Prince Edward Islands (Shabangu, Munoz, et al. 2024).

The May–October throughout the day vocalisation of Antarctic minke whales on and around Child's Bank contrasts with the greater nighttime vocalisation previously observed off the west coast of South Africa (Shabangu, Findlay, and Stafford 2020) and in the sub‐Antarctic region (Shabangu, Munoz, et al. 2024)—this is potentially another special adaptation to the local environment. In the sub‐Antarctic region, killer whales were more vocally active during the day (Shabangu, Daniels, et al. 2024) while Antarctic minke whales vocalised less by day than night (Shabangu, Munoz, et al. 2024), likely as a cryptic response to the presence of killer whales (Branch 2025). In the current study, Antarctic minke whales vocalised from after midnight through to late afternoon in the absence of killer whales (which were not detected), similar to daytime vocalisation observed for this species in Antarctica by Shabangu, Findlay, and Stafford (2020). Furthermore, the lower nighttime vocalisation in June and July could be indicative of nighttime foraging of this species in relationship to their prey diel vertical migration (e.g., Cade et al. 2023). The above observed diel vocalising patterns are also supported by the RF model acoustic occurrence results (Figure 6a). Of interest was that minke‐like whale calls were only detected during the day, similar to the June and July Antarctic minke whale diel vocalising pattern observed in this study.

### 4.4 Acoustic Occurrence Predictors

According to RF models, SSH was the most important predictor of the acoustic occurrence of Antarctic minke and Bryde's whales, whereas SSH and SST were the most important predictors of southern right whale acoustic occurrence. This suggests that seasonal changes in the environmental variables have an important influence on the acoustic occurrence of these marine mammals. This strong link between the acoustic occurrence of these baleen whales and changes in environmental conditions indicates that the area in and around Child's Bank provides a suitable habitat for these whales. SST was the most important predictor of humpback whale acoustic occurrence, likely indicating that the presence and migration patterns of this species coincided with colder environments. The acoustic occurrence of both humpback whales and Antarctic minke whales decreased with increasing SST, as previously shown at the Prince Edward Islands (Shabangu, Munoz, et al. 2024). This may indicate that their timing of migration and movement corresponded with season‐induced changes in SST. The association with cooler waters suggests that whale occurrence may be timed with the start of the upwelling season off the west coast of South Africa. The acoustic occurrence of Bryde's whales increased with SST, supporting that they are partial to slightly warmer waters (e.g., Purdon, Shabangu, Yemane, et al. 2020). Bryde's whales generally have a preference for offshore locations in winter (Purdon, Shabangu, Yemane, et al. 2020) where waters are warmer compared to inshore areas (Andrews and Hutchings 1980; Hutchings et al. 2009).

Antarctic minke and Bryde's whale acoustic occurrence decreased with increasing wind speed, potentially reflecting that wind‐induced noise could be masking the detection of whale sounds (Shabangu et al. 2022) while wind‐generated air bubbles close to the sea surface could have attenuated some whale sounds (e.g., Shabangu et al. 2014). Southern right whale probability of acoustic occurrence increased with wind speed, reflecting that their occurrence coincided with stronger wind speeds associated either with winter storms or, in spring–summer, strong southerly winds that drive upwelling in this region (Andrews and Hutchings 1980; Hutchings et al. 2009). Alternatively, these southern right whales could have been exhibiting Lombard response to wind‐induced underwater noise (e.g., Parks et al. 2011), or they were close to the recorder location to increase their chances of detection. Chlorophyll‐a was a moderately to least important predictor of whale acoustic occurrence, likely reflecting a spatial mismatch between phytoplankton and zooplankton/fish (baleen whale food) abundance in this region of the Benguela ecosystem (Grémillet et al. 2008).

### 4.5 Conclusions

This study underscores passive acoustic monitoring technique as an effective means of determining the occurrence and behaviour of whales, particularly in regions with limited or no dedicated cetacean research taking place. We provide the first quantitative details of the characteristics of the southern African Bryde's whale offshore population. No long‐term acoustic studies on seasonal occurrence previously existed for Bryde's whales in the southern African subregion, and this study provides the first details of acoustic occurrence and highlights the potential to use this method to differentiate the two populations. Studying Antarctic baleen whale species such as Antarctic minke whales in the low latitudes, including South Africa, serves as a cost‐effective manner of studying these species without having to bear the costly effort of traveling to Antarctica. More bioacoustics research is needed to identify the source of the minke‐like whale call detected in this study. Acoustic occurrence and diel vocalising patterns of baleen whales established here advance our understanding of the seasonal occurrence, movement, and vocalising behaviour of these whales in the Southern Hemisphere. Environmental variables were shown to influence the acoustic occurrence of these whales, and continued simultaneous environment and acoustic data collections will be essential for tracking the adaptation of these marine mammals to the changing oceans. This paper bridges the disciplines of marine biology, environmental science, and acoustics, highlighting interdisciplinary approaches crucial for marine conservation.

## Author Contributions

**Fannie W. Shabangu:** conceptualization (equal), data curation (lead), formal analysis (lead), investigation (equal), methodology (equal), software (lead), visualization (lead), writing – original draft (lead). **Stephen P. Kirkman:** conceptualization (equal), funding acquisition (equal), investigation (equal), methodology (equal), project administration (equal), supervision (equal), writing – review and editing (equal). **Kuhle Hlati:** conceptualization (equal), funding acquisition (equal), investigation (equal), methodology (equal), project administration (equal), resources (equal), writing – review and editing (supporting). **Marcel A. van den Berg:** investigation (equal), methodology (equal), project administration (equal), resources (equal), writing – review and editing (supporting). **Tarron Lamont:** funding acquisition (equal), investigation (equal), methodology (equal), project administration (equal), resources (equal), writing – review and editing (equal).

## Disclosure

Animal ethics and welfare: There are no animal ethics and welfare concerns.

## Conflicts of Interest

The authors declare no conflicts of interest.

## Data Availability

Data used for this manuscript can be found through the following doi: https://doi.org/10.5281/zenodo.15593833.
